# Management of burnout among the staff of primary care centres in Spain during the pandemic caused by the SARS-CoV-2

**DOI:** 10.1186/s12960-021-00679-9

**Published:** 2021-11-01

**Authors:** Isaac Aranda-Reneo, Azucena Pedraz-Marcos, Montserrat Pulido-Fuentes

**Affiliations:** 1 Department of Economic Analysis and Finances, Faculty of Social Sciences, Avda. Real Fábrica de Seda S/N, University of Castilla-La Mancha, 45600 Talavera de la Reina, Spain; 2 Nursing Department, Faculty of Medicine, Autonomous University of Madrid, Madrid, Spain; 3 Department of Nursing, Physiotherapy and Occupational Therapy, Faculty of Health Sciences, University of Castilla-La Mancha, Talavera de la Reina, Spain

**Keywords:** Burnout, COVID-19, SARS-CoV-2, Primary care, Workload, Mental health, BCSQ-36

## Abstract

**Background:**

The provision of healthcare during the pandemic caused by the SARS-CoV-2 virus represented a challenge for the management of the resources in the primary care centres. We proposed assessing burnout among the staff of those centres and identifying factors that contributed to its appearance and those that limited it.

**Methods:**

An observational study which, by means of anonymous questionnaires, collected information about: (i) demographic variables; (ii) the characteristics of each position; (iii) the measures implemented by the medical decision-makers in order to provide care during the pandemic; and (iv) the Burnout Clinical Subtype Questionnaire (BCSQ-36). We performed a descriptive analysis of the burnout mentioned by the staff, and, by means of a multivariate analysis, we identified the factors which influenced it. Using logit models, we analysed whether receiving specific training in COVID-19, feeling involved in decision-making processes, and/or working within different healthcare systems had effects on the development of burnout.

**Results:**

We analysed the replies of 252 employees of primary care centres in Spain with an average age of 45 (SD = 15.7) and 22 (SD = 11.4) years of experience. 68% of the participants (*n* = 173) indicated burnout of the frenetic subtype. 79% (*n* = 200) of the employees had high scores in at least one burnout subtype, and 62% (*n* = 156) in at least two. Women older than 45 had a lower probability of suffering burnout. Receiving specific training (OR = 0.28; CI95%: 0.11–0.73) and feeling involved in decision-making (OR = 0.32; CI95%:0.15–0.70) each reduced the probability of developing burnout. Working in a different department increased the likelihood of developing burnout of at least one clinical subtype (OR = 2.85; CI95%: 1.38–5.86).

**Conclusions:**

The staff in primary care centres have developed high levels of burnout. Participation in decision-making and receiving specific training are revealed as factors that protect against the development of burnout. The measures taken to contain the adverse effects of a heavy workload appear to be insufficient. Certain factors that were not observed, but which are related to decisions taken by the healthcare management, appear to have had an effect on the development of some burnout subtypes.

**Supplementary Information:**

The online version contains supplementary material available at 10.1186/s12960-021-00679-9.

## Background

In the management of the pandemic caused by SARS-CoV-2, the Primary Healthcare Service has a fundamental role in the early detection of cases, the tracing of their contacts, the isolation of infected people and of their close contacts and adds to our knowledge about the progress of the disease and the protection of those who look after the affected people [1]. As the “main gateway” to the healthcare system, the professional primary care staff have to take part in planning and acting to manage the risk of health emergencies [2]. The WONCA (World Organization of National Colleges, Academies and Academic Associations of General Practitioners/Family Physicians) has actively upheld the ways in which the primary care service can be supported in its provision of care during emergencies [3].

In Spain, the health system is characterised by the strength of primary care, which is the core element of the health system. Primary care is essentially provided by public providers and is mainly funded by taxes [4]. The central government lays down and coordinates the rules governing the rights of access to each type of medical service but does not specify how those services should be provided. However, the Regions by the Departments of Health decide and plan how to organise the healthcare resources. During the first wave of COVID-19, the Regions imposed similar restrictions on access to their healthcare services (access to the primary care centre was not permitted). They opted either for a system of “remote” care or for a system with restricted care and follow-up of patients [5].

Healthcare workers regard their activity as truly satisfying and gratifying, and sometimes interesting, instructive and agreeable. However, the COVID-19 health crisis has added even more stressful factors to those which already existed in the field of healthcare [6–10]. Uncertainty when taking decisions, a lack of resources or of protective equipment, the re-organisation of their duties, and the priority given to patients with COVID-19, have all contributed to breaking the usual rhythm of work of medical staff [11, 12]. It is important to remember that there is evidence of a connection between the well-being of the medical staff and the safety of their patients, and this connection even extends to medical errors [13].

The impact of the pandemic caused by COVID-19 can be seen in people’s emotional state, and not only in the frequent appearance of stressful variables, but also in their duration and intensity [14]. As they pass through the different stages of the general adaptation syndrome, healthcare workers have to contend with an unprecedented degree of acute stress in their workplace, and this is aggravated by a high basal rate of exhaustion [15]. Some studies in hospitals have found prevalence of anxiety and depression that were close to 25%, with a prevalence of stress of 45% [16], figures that were slightly higher in the study of Hummel et al*.* in eight European countries [17]. These disorders have physical manifestations, the most frequent of which is headache [18]. The existence of social and organisational support, sufficient and appropriate protection and information, and a positive atmosphere at work have all been associated with lower levels of psychological disturbance in the hospital environment [19, 20]. In China, which was one of the first countries in which the SARS-CoV-2 was detected, half of the people working in hospitals have suffered from burnout [21]. The medium-term impact of previous pandemics on healthcare staff working within the community is well known: there were changes in their form of care for their patients, greater safety measures were taken, and tests were delayed [22].

The COVID-19 pandemic has seriously hit Spain and its health system (where public bodies mainly deliver primary health care) [23]. Information about the significance of COVID-19 on the Spanish primary care health system can provide valuable insights to public and private decision-makers because of its similarity with other European and non-European national health systems [24]. It is possible that new scenarios, similar to the current one, will appear in the future. Moreover, the pandemic caused by SARS-CoV-2 is not yet completely under control. Then, assessing burnout among the staff of primary care centres and evaluate the management strategies deployed by decision-makers in a high workload scenario could help to prevent the appearance of burnout among healthcare workers placed in the primary care level..

## Methods

We aimed to study the burnout of primary care staff and evaluate the effectiveness of specific actions deployed by decision-makers on the development/prevention of burnout among those workers. We hypothesised that primary health care workers had burnout during the first wave of the COVID-19 pandemic. Moreover, we postulated the actions suggested by decision-makers did not work to improve their working environment. An observational study using anonymous questionnaires distributed via the internet, and which the respondents completed between June and November 2020. Letters of invitation to the study were sent to the e-mail addresses of 677 healthcare staff in primary care centres in two adjoining Spanish regions that had been similarly affected by COVID-19 [25]. Nevertheless, these regions depend on different regional governments with varying strategies concerning the health care system management. These differences in health strategies can be resumed in differences in health care expenditure that range from €1,340 per inhabitant (region A) to €1,501 (region B) in 2019[26]. The respondents agreed to participate in the study, and the project was evaluated positively by local ethical committees.

### Measures

The questionnaires included questions designed to collect information about socio-demographic characteristics (age, sex, marital status, region in which the respondent works), questions about the job (type of contract, length of service, whether a medical or non-medical worker), the means provided by the healthcare department to help with the provision of healthcare during the first wave (whether the respondent participated in decision-making, whether they received specific training in COVID-19), and the Burnout Clinical Subtype Questionnaire (BCSQ-36). The latter had been previously validated in the general population [27] and also in primary-care doctors [28]. The BCSQ-36 includes a Likert scale of 1–7 points to assess the burnout in each clinical subtype, and has demonstrated, in comparison with other tools, validity and reliability for identifying three different subtypes of burnout, ‘frenetic’ ‘underchallenged’ and ‘worn-out’ [29]. The frenetic subtype appears in people who are heavily involved in their work and overburdened by the demands of the job. The ‘underchallenged’ subtype appears in workers who feel that their tasks are excessively monotonous and do not provide them with the satisfaction that they had hoped for. The last subtype, ‘worn-out’, appears when people perceive a lack of acknowledgement of their work, a failure to check their results and/or an abandonment of their responsibilities. The BCSQ-36 has no cutoff points, so, like other authors, we consider as high marks in each subtype those that are above the third quartile [27].

In order to assess what could have contributed the most to the development of burnout among the participants in the study, two groups of variables were distinguished: (i) characteristics of the job, and (ii) elements connected with the healthcare system’s management of resources. The length of service (in years), the type of contract (permanent or temporary) and the occupation (nursing/medicine/other) were regarded as factors related to the job. We considered as factors related to the health system’s management of resources: whether specific training in COVID-19 was provided (yes/no), whether the medical worker participated in decision-making (yes/no), and the region in which he/she worked (Zone A/Zone B).

### Statistical analysis

We performed a descriptive analysis of the scores of the participants in the study in each of the subtypes of burnout identified by the BCSQ-36, using the mean, the median and the values of the first and last quartiles. We also used proportions to show the proportion of participants who reached high scores on the scales of the BCSQ-36 [27]. High percentages signified a large number of people with burnout. We also identified those participants who showed burnout of at least one, and those who showed it in two, of the three subtypes of the BCSQ-36. Thus, we can assess if the prevalence of burnout was only due a one or two subtypes. In this sense, higher number of participants with higher values in two subtypes indicated worse working environment, and therefore, higher harmful effects expected due to work during the pandemic COVID-19. We used the usual contrasts to assess whether statistically significant differences were observed in the proportion of participants with high scores when we took account of the different variables that we considered to be of interest. We also took possible socio-demographic differences into account, as control variables. We performed a multivariate analysis using Logit models and, as dependent variable, the presentation of high scores in any subtype (or high scores in at least one burnout subtype, or in two), and as independent variables, socio-demographic factors related to the job, and elements connected with the management of the pandemic by the national health system. The descriptive analysis was performed with the help of the SPSS statistical program, and the multivariate analysis, with Stata SE.

## Results

We analysed 252 valid responses (37%) to the 677 questionnaires sent out. 57% were from community nurses, 31% were from doctors working in primary care or paediatrics, and 12% were from non-medical staff. The average age was 45 (SD = 15.7), with 22 (SD = 11.4) years of professional experience. 80% were women and 78% were living in a couple or in a marriage at the time of their participation in the project. No statistically significant differences were found between Zones A and B with respect to marital status, years of service, age or type of job, but differences were found in relation to sex. In Zone A, 85% were women, and in Zone B, 71% (*χ*^2^ = 7.3; *p* < 0.01). Table 1 shows the participants characteristics who were included in the study.

**Table 1:** Participants main characteristics

	All respondents (*n* = 252) (%)
Sex	
Female	80
Age	
< 35 years	24
35–48 years	26
> 48 years	50
Size of household	
1 person	14
2 people	31
3–4 people	44
> 4 people	11
Length of service	
< 13 years	25
13–23 years	26
> 23 years	49
Contract duration	
Temporary	44
Permanent	56
Occupation	
NHP	12
Nurse	57
Doctor	31
Specific COVID-19 training provided*	
No	73
Yes	27
Took part in decision-making	
No	61
Yes	39
Zone	
B	37
A	63

NHP non-healthcare professional

*Provided by the National Health System/Health authorities

79% (*n* = 200) of the participants in the study obtained high scores (> = Q_3_) in at least one of the two subtypes of burnout included in the BCSQ-36, 62% (*n* = 156) in at least two and 49% (*n* = 123) of participants reached high scores in the three subtypes. The dimension with the highest percentage of people (68%) with high scores was the frenetic subtype. In the subtype ‘underchallenged’ 27% obtained high scores, and in the subtype ‘worn-out’, 25% did (Table 2). Mean scores obtained on each subtype by participants together with other statistics are provided as Additional file 1.

**Table 2:** Percentage of participants with the highest scores (above the Q_3_) in each burnout subtype by variables of interest

	High score in Frenetic (68% of the sample)		High score in Underchallenged (27% of the sample)		High score in Worn-out subtype (25% of the sample)		High scores in at least 1 subtype. (79% of the sample)		High scores in at least 2 subtypes. (62% of the sample)	
	%	*p* value	%	*p* value	%	*p* value	%	*p* value	%	i value
Sex										
Male	64		30		14		76		54	
Female	70	0.44	26	0.55	28	**0.04**	80	0.52	64	0.2
Age										
< 35 years	80		39		26		89		72	
35–48 years	68		29		22		82		62	
> 48 years	64	0.06	20	**0.02**	26	0.75	74	0.06	57	0.141
Size of household										
1 person	62		35		32		79		65	
2 people	76		30		26		81		67	
3–4 people	69		26		25		78		56	
> 4 people	61	0.34	11	0.15	14	0.43	79	0.97	68	0.43
Length of service										
< 13 years	81		35		24		89		68	
13–23 years	62		31		23		83		66	
> 23 years	66	**0.04**	21	0.09	27	0.84	73	**0.02**	57	0.2
Contract duration										
Temporary	71		34		25		88		69	
Permanent	66	0.353	21	**0.02**	25	0.97	72	**0.03**	56	**0.04**
Occupation										
NHP	72		24		16		76		72	
Nurse	70		25		23		81		59	
Doctor	66	0.76	32	0.56	34	0.09	80	0.86	63	0.42
Specific COVID-19 training provided^a^										
No	70		32		30		83		65	
Yes	65	0.5	13	**0.002**	12	**0.003**	70	**0.02**	54	0.1
Took part in decision-making										
No	66		32		32		79		64	
Yes	73	0.25	19	**0.02**	12	**0.001**	77	0.77	58	0.29
Zone										
B	68		19		13		68		54	
A	69	0.88	32	**0.03**	32	**0.001**	86	**0.001**	67	0.05

Bold text indicates statistical significance

*NHP* non-healthcare professional

^a^Provided by the National Health System/Health authorities

Table 2 shows the percentage of people who had high scores in any of the burnout subtypes studied. We also analysed the percentages of those who had high scores in at least one subtype and in at least two. A significantly higher probability of obtaining high scores in the ‘underchallenged’ dimension was noticed among young participants. Statistical significance was also found on analysing the high scores of both women and men in the ‘worn-out’ subtype, and, here, more women than men had high scores. However, neither sex nor age was significant when high scores were obtained in at least one or in at least two subtypes. The type of job was not a very important factor in high scores in any of the subtypes evaluated by the BCSQ-36. Nor was it a significant factor in burnout of at least one subtype or of two subtypes. However, the amount of professional experience and the duration of the contract did have a significant effect on the percentage of people with high scores in at least one subtype and in two. When the contract was temporary, there were higher percentages of people with high scores in at least one subtype and in two subtypes. Having more professional experience acted as a protective factor. The shorter the lengths of service in a job, the greater the proportion of high scores. Our analysis showed that, compared with socio-demographic variables or variables related to the job, elements related to the management of the pandemic were much more closely associated with the development of burnout. The percentages of people with high scores in the ‘underchallenged’ and ‘worn-out’ subtypes were significantly lower among workers who reported that they had felt involved in making decisions about the pandemic. Having received training in COVID-19 from the health service also had a positive effect, and a lower percentage of people with high scores was observed among people who had received such training. Finally, we found that participants who worked in Zone A were more likely to have high scores in the ‘underchallenged’ and ‘worn-out’ subtypes. In the ‘frenetic’ subtype, we observed no statistically significant differences that varied according to the healthcare service provider for which the participant worked.

The result of the multivariate analysis confirmed that the development of some clinical subtypes of burnout was more strongly associated with the manner of approaching the pandemic than with demographic factors or the job. Workers who reported that they had received training in COVID-19 had a lower risk of obtaining high scores in the ‘underchallenged’ subtype than participants who had received no such training (Table 3). This positive effect was maintained when workers in this same ‘underchallenged’ subtype themselves obtained and accumulated training in COVID-19 (OR = 0.20; CI95%: 0.07–0.54) (Additional file 2). Moreover, when the workers themselves obtained this training there was also a lower probability of significant numbers of them obtaining high scores in the ‘worn-out’ subtype (OR = 0.31; CI95%: 0.12–0.79) (Additional file 2), an effect that was not observed when the training was provided by the national health system (Table 3); and this had statistical significance as a factor protecting against the development of burnout in at least one of the three subtypes (OR = 0.4; 95%CI: 0.19–0.86) and in at least two (OR = 0.51; 95%CI: 0.27–0.99) (Additional file 2). Participation in the decision-making processes also reduced the probability of developing burnout in the ‘underchallenged’ subtype and of showing high scores in the ‘worn-out’ subtype. However, working in Zone A significantly increased the risk of obtaining high scores in this subtype, and also increased the probability of reaching high scores in at least one of the three subtypes (Table 3).

**Table 3:** Risk factor analysis of each burnout subtype

	High score in Frenetic		High score in Underchallenged		High score in Worn-out subtype		High scores in at least 1 subtype		High scores in at least 2 subtypes	
	OR	CI 95%	OR	CI 95%	OR	CI 95%	OR	CI 95%	OR	CI 95%
Sex										
Male (ref)										
Female	1.19	0.56–2.55	**0.36**	**0.16–.83**	1.71	0.66–4.45	0.92	0.38–2.23	0.99	0.48–2.04
Age										
< 35 years (ref)										
35–48 years	0.69	0.19–2.53	0.60	0.18–1.98	1.05	0.29–3.82	1.67	0.36–7.82	0.67	0.22–2.09
> 48 years	0.38	0.10–1.39	**0.21**	**0.06–0.75**	1.03	0.29–3.70	1.22	0.31–4.82	0.65	0.20–2.04
Size of household										
1 person (ref)										
2 people	1.71	0.64–4.56	0.65	0.23–1.89	0.97	0.33–2.85	1.11	0.36–3.45	0.99	0.39–2.54
3–4 people	1.42	0.57–3.55	0.80	0.29–2.16	1.04	0.37 –2.88	1.33	0.45–3.93	0.84	0.34–2.06
> 4 people	1.28	0.39–4.20	0.33	0.07–1.53	0.61	0.14–2.58	2.01	0.48–8.46	2.10	0.62–7.15
Length of service										
< 13 years (ref)										
13–23 years	0.45	0.12–1.69	1.60	0.45–5.78	1.05	0.27–4.15	0.42	0.07–2.36	1.15	0.34–3.81
> 23 years	0.87	0.19–3.98	1.49	0.32–6.89	1.09	0.22–5.42	0.33	0.06–2.00	1.01	0.25–4.07
Contract duration										
Temporary (ref)										
Permanent	1.01	0.39–2.62	1.23	0.42–3.61	1.37	0.44–4.26	0.67	0.22–2.03	0.78	0.31–2.00
Occupation										
NHP (ref)										
Nurse	0.69	0.25–1.97	0.77	0.25–2.38	1.64	0.47–5.79	1.36	0.43–4.33	0.52	0.19–1.44
Doctor	0.77	0.26 –2.32	0.98	0.30–3.22	2.56	0.70–9.34	1.19	0.35–4.07	0.62	0.21–1.82
Specific COVID-19 training provided^a^										
No (ref)										
Yes	1.40	0.67–2.92	**0.28**	**0.11**–**0.73**	0.42	0.17–1.06	0.59	0.28–1.28	0.76	0.39–1.48
Took part in decision-making										
No (ref)										
Yes	1.46	0.77–2.75	**0.45**	**0.22**–**0.94**	**0.32**	**0.15**–**0.70**	1.15	0.56–2.36	0.86	0.48 –1.53
Zone										
B (ref)										
A	1.07	0.56–2.06	1.38	0.65–2.93	**2.49**	**1.11**–**5.59**	**2.85**	**1.38**–**5.86**	1.59	0.86–2.91

Bold text indicates statistical significance

*NHP* non-healthcare professional

^a^Provided by the National Health System/Health authorities

## Discussion

Our task was to study the appearance of burnout among primary care staff during the pandemic caused by SARS-COV-2, and to assess whether the decisions taken by the administrators as a result of the pandemic had any effect on the development of burnout. The population included in our study suffered very high levels of burnout in their jobs. Compared with the results of other studies that were also carried out on primary care workers, but before the pandemic, the average scores obtained in our study were higher for all the subtypes of burnout [28]. The management strategies implemented by the medical decision-makers (participation in decision-making, provision of training in COVID-19) had effects that were more important than those of the factors related to the job (duration of the contract, years of experience) or to the socio-demographic characteristics of the worker (sex, age).

Other authors have recently assessed the effects on healthcare workers of the heavy workload resulting from COVID-19. However, there is a lack of evidence that reveals the effects of the pandemic outside the hospital setting [30]. Most of the COVID-19 related studies have focused on clinical symptoms, vaccine development or detection methods [31]. Luceño-Moreno et al. carried out a study of medical personnel in hospitals, day care centres, nursing homes and primary care centres. The data were collected during April, 2020, and these authors discovered that 82% of the participants in the study had high scores in the ‘personal accomplishment’ dimension and 41% in the ‘emotional exhaustion’ dimension of the Maslach Burnout Inventory. Our figures are similar to those obtained by the authors mentioned, but slightly lower. Nevertheless, authors indicated that the figures for anxiety and depression could have been overestimated because of the moment when the data were collected (a lot of uncertainty, a lack of knowledge and a shortage of equipment for personal protection) [32]. Torrente et al. evaluated the prevalence of burnout among healthcare workers during 2 weeks immediately following the first peak of the pandemic. These authors estimated that the prevalence of burnout reached 43.4%, and showed statistically significant differences between workers dealing directly with COVID patients (50%) and those working with non-COVID patients (35%). However, of the population included in their study, only 10% worked in primary care, and no distinction could be shown in the results corresponding to the different levels of care provided [33]. In our study, we did not record whether the participants worked directly with COVID-19 patients, but in Spain generally, the tracing and monitoring of contagion was being carried out in the primary care centres, though this did not result in an absence of care for patients with other diagnoses. In contrast, in the hospitals, surgical operations have been discontinued and oncological treatments have been postponed [12, 34, 35]. Therefore, the higher prevalence of burnout in our population could be due to the fact that in the primary care centres the caring work has not been completely halted. Ruiz-Fernández et al. interviewed, on line, healthcare workers at different levels, with the aim of assessing the amount of stress that these people noticed during the first few months of the pandemic, and also their quality of life. The percentage of workers in primary care centres was a little higher (37%) than that covered by other authors [32, 33], but even so, more than 50% of the participants were in a hospital environment, and evaluated the level of burnout by means of a scale which rated the quality of professional life (ProQol) [36]. In this case the percentage of workers with burnout was 36%, which was a little lower than that identified by our own study [37]. However, the authors of this study found statistically significant differences in the presence of burnout among workers at different levels of care, obtaining higher values in hospital environments. Our study collected data after the first wave of the illness, using a tool that had been validated for identifying the syndrome of burnout in this population [28], so we believe that we recorded the situation in the population of the study more accurately, and that the fact that our figures are higher is completely understandable. Finally, Mira et al. used a new tool which was specifically designed to record stress resulting from working with patients diagnosed as having COVID-19 [11]. In their study, 82% of the participants worked in hospitals and only 8% worked in primary care centres. The authors indicated that 5% of the participants in the study suffered extreme levels of stress through caring for patients diagnosed as having COVID-19, but they also indicated statistically significant differences depending on the number of deaths in the area due to COVID-19. They found differences of up to 30% in the scores, which were significantly higher in regions recording a greater number of deaths. In our study, the region in which the medical professional was working at the time of the online interview proved to be a significant factor, and one which increased the probability of developing burnout of the ‘worn-out’ subtype. However, in Zone A prevalence of COVID-19 of 11.5% was revealed, whereas in Zone B the prevalence was of 11.1% [25], so we believe that exposure to the effects of the pandemic is not the only reason for the greater risk of developing burnout in one zone than in another. Indeed, the differences in the average scores for the ‘underchallenged’ and ‘worn-out’ subtypes were 0.7 (CI95%: −0.74, −0.11) and 0.42 (CI95%: −0.93, −0.46) less in Zone B, and in the frenetic subtype the difference was 0.06 (CI95%: −0.18, 0.3) more in Zone B.

Our study found that the effects of the management strategies implemented by the healthcare services were more important than effects related to the job or to the socio-demographic characteristics which had previously been thought to be connected with cases of more serious burnout [38]. Specifically, workers who felt that they had taken part in making decisions about the pandemic, and had received training in COVID-19 from the health service, had lower scores for burnout. We also noticed that when training in COVID-19 was obtained individually, the protective effect was greater than when the training was provided by the national health system. It is, therefore, very important to provide staff with carefully prepared training sessions, and it is essential in demanding circumstances. Reverting to the question of workers’ participation in decision-making, in other studies it has been found to be a positive factor, but also a factor that itself produces stress. Pollock et al., in a systematic review of the types of intervention that effectively protect workers’ mental health during pandemics, draw attention to the importance of creating feelings of security and connection through interventions such as those that give reliable information and those that facilitate the exchange of experiences and the participation of staff in organisational tasks [39]. In contrast, in the study of González et al., nurses in the intensive care and emergencies departments, who had been given more autonomy in making decisions about the use of medicines, experienced discomfort, because those decisions had to be taken in situations of increasing workload, scarcity of resources and difficulties in communicating with middle management [19]. On the other hand, receiving training in COVID-19 had the effect of reducing the probability of obtaining high scores for burnout in two of the three subtypes. That effect remained even when it was the workers themselves who had obtained this specific training, and it also reduced the probability of experiencing burnout of the ‘worn-out’ subtype (OR = 0.32; CI95% = 0.12–0.79). In the literature, there has been a greater consensus about the importance of training as an element that protects against burnout during a pandemic [20]. This means structured training, even online training [40], but also information and clear guides to action to deal with infection and the risk of contagion [18, 19, 41]. Training in tools for managing psychological risks has also been regarded as fundamental during this pandemic [42].

Among the principal limitations of the study has been the high rate of failure to respond. This has also been mentioned by other authors who used surveys before the pandemic [38]. Medical activity in the primary care centres has not ceased during the pandemic, and the care of patients with other diagnoses has continued, even when there has been a lot of absence from work due to infection of the staff by COVID-19. Moreover, a lot of research has been begun in this same area and may have led to a feeling of saturation among medical staff who were asked to take part in the study. On the other hand, our study has a transverse design, whereas studies with longitudinal designs may not always find the relationships that we find between the risk factors and the development of burnout. Finally, the participants who completed the survey may have been those who felt the worst, in which case we may have been overestimating the gravity of the real situation. Nevertheless, as far as we know, this study is the first that has explored the effect of the pandemic exclusively on primary care workers. Moreover, the tool that we used had been previously validated for the diagnosis of burnout in that population, and it is also the first to explore the effect on the development of burnout of the measures taken by the administrators to deal with the workload of those people resulting from the health crisis caused by the pandemic. New work is needed to study these effects in greater detail, both to assess whether there are better ways of tackling the problem of burnout and to discover whether the presence of burnout among these workers is likely to endure. The healthcare administrators should monitor the effects of burnout on the worsening of the health of their staff, not only because those workers’ own health is at risk, but also because an association between the well-being of this population and the safety of the patient has already been revealed [13].

## Conclusions

The staffs of primary care centres have been exposed to workloads that have caused them to develop burnout. Some of the strategies proposed by the healthcare administrators have had positive effects which may have avoided a greater adverse effect of the heavy workload which these professionals had to bear to fulfil the demands of their jobs. However, there are apparently some variables and decisions, not observed in our study, but which would be directly related to certain measures, proposed by the healthcare authorities, which do not contribute to a containment of the adverse effects of the overworking that is now required in these primary care centres.

## Supplementary Information

**Additional file 1: Table S1.** Descriptive statistics for the BCSQ-36 scale (n = 252).

**Additional file 1: Table S2.** Risk factor analysis of each burnout subtype.

## Data Availability

The data sets used and/or analysed during the current study are available from the corresponding author on reasonable request.

## Abbreviations

SARS-CoV-2: Severe acute respiratory syndrome coronavirus 2
WONCA: World Organization of National Colleges, Academies and Academic Associations of General Practitioners/Family Physicians
COVID-19: Coronavirus disease 2019
BCSQ-36: Burnout Clinical Subtype Questionnaire
OR: Odd ratio
SD: Standard deviation
NHP: Non-healthcare professional
ProQol: Professional Quality of Life
