# Supplementary material for: Management of burnout among the staff of primary care centres in Spain during the pandemic caused by the SARS-CoV-2
Source: Hum Resour Health. 2021 Nov 1;19:133. doi: 10.1186/s12960-021-00679-9 (PMC8558543; doi:10.1186/s12960-021-00679-9)
Supplement: Supplementary file 1 — Additional file 1: Table S1. Descriptive statistics for the BCSQ-36 scale (n = 252). [file 12960_2021_679_MOESM1_ESM.docx]

Additional file 1: Descriptive statistics for the BCSQ-36 scale (n=252).

|  | Mean | SD | Q1 | Q3 | min | max |
| --- | --- | --- | --- | --- | --- | --- |
| Frenetic subtype | 4.37 | 0.92 | 3.83 | 5.00 | 1.00 | 6.83 |
| Involvement | 5.34 | 0.91 | 5.00 | 6.00 | 1.00 | 7.00 |
| Ambition | 3.93 | 1.28 | 3.00 | 5.00 | 1.00 | 7.00 |
| Overload | 3.84 | 1.32 | 3.00 | 4.75 | 1.00 | 7.00 |
| Underchallenged subtype | 2.78 | 1.23 | 1.83 | 3.50 | 1.00 | 6.83 |
| Indifference | 2.46 | 1.12 | 1.5 | 3.00 | 1.00 | 6.50 |
| Boredom | 2.86 | 1.40 | 1.75 | 3.63 | 1.00 | 7.00 |
| Lack of Development | 3.03 | 1.52 | 1.75 | 3.75 | 1.00 | 7.00 |
| Worn-out subtype | 3.65 | 0.96 | 3.08 | 4.21 | 1.00 | 6.00 |
| Lack of control | 4.46 | 1.26 | 3.50 | 5.25 | 1.00 | 7.00 |
| Lack of Acknowledgement | 4.30 | 1.41 | 3.25 | 5.25 | 1.00 | 7.00 |
| Neglect | 2.19 | 0.88 | 1.25 | 3.00 | 1.00 | 4.75 |
