# Supplementary material for: Management of burnout among the staff of primary care centres in Spain during the pandemic caused by the SARS-CoV-2
Source: Hum Resour Health. 2021 Nov 1;19:133. doi: 10.1186/s12960-021-00679-9 (PMC8558543; doi:10.1186/s12960-021-00679-9)
Supplement: Supplementary file 2 — Additional file 1: Table S2. Risk factor analysis of each burnout subtype. [file 12960_2021_679_MOESM2_ESM.docx]

Additional file 2: Risk factor analysis of each burnout subtype.

|  | High score on Frenetic | | High score on Underchallenged | | High score on Worn-out sub-type | | High scores on at least 1 sub-type. | | High scores on at least 2 sub-types. | |
| --- | --- | --- | --- | --- | --- | --- | --- | --- | --- | --- |
|  | OR | CI 95% | OR | CI 95% | OR | CI 95% | OR | CI 95% | OR | CI 95% |
| Gender |  |  |  |  |  |  |  |  |  |  |
| Male (ref) |  |  |  |  |  |  |  |  |  |  |
| Female | 1.15 | 0.54– 2.43 | 0.37 | 0.16 –0.85 | 1.72 | 0.66 – 4.47 | 1.00 | 0.42 – 2.37 | 1.01 | 0.49 – 2.07 |
| Age |  |  |  |  |  |  |  |  |  |  |
| <35 years (ref) |  |  |  |  |  |  |  |  |  |  |
| 35 – 48 years | 0.65 | 0.18 – 2.40 | 0.77 | 0.23 – 2.63 | 1.29 | 0.35 – 4.82 | 2.06 | 0.42 – 10.04 | 0.77 | 0.24 – 2.43 |
| > 48 years | 0.36 | 0.10 – 1.32 | 0.26 | 0.08 – 0.9 | 1.24 | 0.35 – 4.47 | 1.37 | 0.33 – 5.61 | 0.68 | 0.21 – 2.19 |
| Size of household |  |  |  |  |  |  |  |  |  |  |
| 1 person (ref) |  |  |  |  |  |  |  |  |  |  |
| 2 people | 1.78 | 0.67 – 4.74 | 0.58 | 0.2 – 1.69 | 0.89 | 0.3 – 2.62 | 0.99 | 0.31 – 3.12 | 0.93 | 0.36 – 2.42 |
| 3 – 4 people | 1.46 | 0.58 – 3.69 | 0.68 | 0.24 – 1.9 | 0.96 | 0.34 – 2.73 | 1.19 | 0.39 – 3.60 | 0.78 | 0.31 – 1.94 |
| > 4 people | 1.37 | 0.42 – 4.48 | 0.23 | 0.05 – 1.1 | 0.49 | 0.11 – 2.12 | 1.66 | 0.39 – 7.11 | 1.9 | 0.56 – 6.51 |
| Length of service |  |  |  |  |  |  |  |  |  |  |
| < 13 years (ref) |  |  |  |  |  |  |  |  |  |  |
| 12 – 23 years | 0.46 | 0.12 – 1.73 | 1.51 | 0.41 – 5.50 | 0.95 | 0.24 – 3.79 | 0.42 | 0.07 – 2.44 | 1.12 | 0.33 – 3.79 |
| > 23 years | 0.9 | 0.2 – 4.07 | 1.33 | 0.29 – 6.17 | 0.96 | 0.19 – 4.81 | 0.33 | 0.05 – 2.03 | 1.00 | 0.25 – 4.10 |
| Contract duration |  |  |  |  |  |  |  |  |  |  |
| Temporary (ref) |  |  |  |  |  |  |  |  |  |  |
| Permanent | 1.03 | 0.4 – 2.68 | 1.08 | 0.36 – 3.25 | 1.28 | 0.41 – 4.04 | 0.61 | 0.20 – 1.88 | 0.74 | 0.29 – 1.91 |
| Occupation |  |  |  |  |  |  |  |  |  |  |
| NHP (ref) |  |  |  |  |  |  |  |  |  |  |
| Nurse | 0.67 | 0.24 – 1.91 | 0.88 | 0.28 – 2.74 | 1.9 | 0.53 – 6.74 | 1.53 | 0.47 – 4.99 | 0.55 | 0.19 – 1.55 |
| Doctor | 0.71 | 0.24 –2.17 | 1.35 | 0.41 – 4.46 | 3.42 | 0.92 – 12.71 | 1.49 | 0.42 – 5.3 | 0.7 | 0.23 – 2.12 |
| Specific Covid-19 training provided* |  |  |  |  |  |  |  |  |  |  |
| No (ref) |  |  |  |  |  |  |  |  |  |  |
| Yes | 1.40 | 0.68 – 2.92 | 0.20 | 0.07 – 0.54 | 0.31 | 0.12 – 0.79 | 0.4 | 0.19 – 0.86 | 0.51 | 0.27 – 0.99 |
| Took part in decision-making |  |  |  |  |  |  |  |  |  |  |
| No (ref) |  |  |  |  |  |  |  |  |  |  |
| Yes | 1.45 | 0.77 – 2.74 | 0.47 | 0.23 – 0.98 | 0.32 | 0.15 – 0.69 | 1.19 | 0.58 – 2.47 | 0.87 | 0.48 –1.56 |
| Zone |  |  |  |  |  |  |  |  |  |  |
| B (ref) |  |  |  |  |  |  |  |  |  |  |
| A | 1.05 | 0.55 – 2.00 | 1.52 | 0.71 – 3.25 | 2.53 | 1.12 – 5.72 | 2.75 | 1.34 – 5.63 | 1.52 | 0.83 – 2.76 |

NHP=non-healthcare professional. *Provided by her/his own.
